# Peptide Nanosheet-Inspired Biomimetic Synthesis of CuS Nanoparticles on Ti_3_C_2_ Nanosheets for Electrochemical Biosensing of Hydrogen Peroxide

**DOI:** 10.3390/bios13010014

**Published:** 2022-12-22

**Authors:** Danzhu Zhu, Hao Kong, Guozheng Yang, Peng He, Xin Luan, Lei Guo, Gang Wei

**Affiliations:** 1 College of Chemistry and Chemical Engineering, Qingdao University, Qingdao 266071, China; 2 Institute of Biomedical Engineering, College of Life Science, Qingdao University, Qingdao 266071, China

**Keywords:** biomimetic synthesis, Ti_3_C_2_ nanosheets, CuS nanoparticles, electrochemical biosensor, H_2_O_2_

## Abstract

Hydrogen peroxide (H_2_O_2_) is one of the intermediates or final products of biological metabolism and participates in many important biological processes of life activities. The detection of H_2_O_2_ is of great significance in clinical disease monitoring, environmental protection, and bioanalysis. In this study, Ti_3_C_2_-based nanohybrids are prepared by the biological modification and self-assembled peptide nanosheets (PNSs)-based biomimetic synthesis of copper sulfide nanoparticles (CuS NPs), which show potential application in the fabrication of low-cost and high-performance electrochemical H_2_O_2_ biosensors. The synthesized CuS-PNSs/Ti_3_C_2_ nanohybrids exhibit excellent electrochemical performance towards H_2_O_2_, in which CuS NPs can catalyze the decomposition of H_2_O_2_ and realize the transformation from a chemical signal to an electrical signal to achieve the purpose of H_2_O_2_ detection. The prepared CuS-PNSs/Ti_3_C_2_-based electrochemical biosensor platform exhibits a wide detection range (5 μM–15 mM) and a low detection limit (0.226 μM). In addition, it reveals good selectivity and stability and can realize the monitoring of H_2_O_2_ in a complex environment. The successful biomimetic synthesis of CuS-PNSs/Ti_3_C_2_ hybrid nanomaterials provides a green and friendly strategy for the design and synthesis of functional nanomaterials and also provides a new inspiration for the construction of highly effective electrochemical biosensors for practical detection of H_2_O_2_ in various environments.

## 1. Introduction

As a very important biomolecule that is related to biological reaction processes, hydrogen peroxide (H_2_O_2_) is of great importance in food science, industrial production, clinical medicine, and biological life activities. In particular, H_2_O_2_ is a very important substance involved in the metabolism of living organisms, but excessive accumulation of H_2_O_2_ can cause irreparable damage to cells and living organisms [1,2]. Therefore, the level of H_2_O_2_ has become an important parameter to determine whether the cells are normal or not, and the detection of H_2_O_2_ has become a key factor in the early diagnosis of some diseases. Among various methods for the detection of H_2_O_2_, electrochemical detection has the characteristics of being a simple operation and having a low detection limit, high sensitivity, and good selectivity; therefore, it has great potential for the detection of H_2_O_2_ with high sensing performance [3,4,5,6]. In order to fabricate high-performance electrochemical H_2_O_2_ biosensors, it is necessary to find active materials with good electrochemical properties.

The easy surface modification, large specific surface area, and unique layered structure of two-dimensional (2D) materials have tremendous advantages for the design and fabrication of electrochemical sensors and biosensors [7,8,9,10,11]. As one of the emerging 2D materials, MXene is mainly composed of layered transition metal carbides or nitrides. MXene has shown powerful capabilities in catalysis, biosensing, capacitors, and electrochemistry by virtue of its large specific surface area, potential surface modifiability, good biocompatibility, excellent electrical conductivity, and high electron transfer effects [12,13,14]. Compared with graphene, which opens the door for 2D materials, Ti_3_C_2_ MXene materials with faster electron mobility are endowed with enhanced electrical conductivity, and studies have been conducted using Ti_3_C_2_ nanohybrids for various electrochemical sensors. For example, Ti_3_C_2_ has been modified with platinum nanoparticles (Pt NPs) for electrochemical detection of H_2_O_2_. The Ti_3_C_2_/Pt NPs-modified glassy carbon electrode (GCE) exhibited a stronger redox capability and also revealed a lower detection limit and better stability for the detection of H_2_O_2_ [15]. Therefore, it is important to take advantage of the electrochemical properties of Ti_3_C_2_ in combination with other materials to increase the abundance of Ti_3_C_2_ surface groups, in order to further improve the biocompatibility and electrochemical properties of Ti_3_C_2_ nanosheets [16].

Peptide is a kind of widely used biomolecule with a tailored self-assembly function, which consists of amino acids that are arranged in a certain sequence. The amino and carboxyl groups at the head and tail (or on the side chains) of peptides are able to complex and coordinate with metals or metal oxides, thus enhancing the biocompatibility of metals. It has been demonstrated that metal ions play a very important role in the self-assembly process of some peptides [17,18]. For instance, Vello et al. studied the reciprocal recognition of amyloid-β peptide (Aβ) by Zn(II) and Cu(II) ions and found that Aβ fibrils encapsulated with Cu(II) were able to generate reactive oxygen species, which can lead to cell death in the presence of H_2_O_2_ and reducing agents [19]. Additionally, the charge carried by the peptide changes with the pH of the solution system. The pH of the peptide in electroneutrality is called its isoelectric point, and this property enables it to adsorb metal ions through electrostatic interactions under the modulation of the external environment. For instance, in the work of Liu et al., a peptide with the isoelectric point of 9.94 was used to adsorb Ag^+^ in a neutral environment, and then Na_2_S was used for the in-situ reduction of Ag^+^ on the peptides to form Ag_2_S NPs for photothermal therapy of tumors [20]. Peptide-based nanomaterials with good morphology and functionality can be easily obtained by designing the peptide sequence and controlling the self-assembly conditions, which can be further used as a bridge between materials to construct hybrid nanomaterials with unique structures and desirable properties [21,22,23,24].

In the conjugation process of peptides with 2D materials, the cross-linking agents are usually required to achieve the binding. Glutaraldehyde (GA) is a widely used biological cross-linking agent whose aldehyde groups can react with -OH or -NH_4_ groups to achieve the cross-linking. In the work of Ou et al., the binding of GO with cellulose was achieved by GA-based linking, where both GO and cellulose surfaces are rich in the presence of -OH groups, and where GA binds to the -OH groups on the cellulose surface at one end and to the -OH group on the GO surface at the other end [25]. Besides reacting with the -OH group, GA is also capable of cross-linking with the -NH_4_ group. The surface of Ti_3_C_2_ that is synthesized by the etching method is rich in the -OH group, which can react with the aldehyde group of GA, while the aldehyde group at the other end of GA can react with the -OH group or the -NH_4_ group of peptide molecules to realize the combination. Therefore, GA-modified Ti_3_C_2_ will widen the distance between Ti_3_C_2_ nanosheets and narrow the force between nanosheets, making Ti_3_C_2_ have better dispersion and stability.

In this work, GA is used as a cross-linking agent to connect Ti_3_C_2_ with self-assembled peptide nanosheets (PNSs) to enrich the Ti_3_C_2_ surface groups. The peptide with the sequence KLVFFAK is selected to modify the Ti_3_C_2_ surface for this aim. The peptide sequence KLVFFAK is derived from a partial fragment of β-amyloid and is able to form nanosheets of moderate thickness and size through π–π interactions [26,27,28]. By modulating the self-assembly of KLVFFAK peptides, smaller size PNSs are prepared and modified onto the Ti_3_C_2_ surface by GA-based cross-linking, which leads to an increase in the type and number of groups on the Ti_3_C_2_ surface. At the same time, the isoelectric point of the KLVFFAK peptide is 10.6, and the PNSs formed by the peptide self-assembly under neutral and acidic environments have a negative charge, which enhances the electronegativity of Ti_3_C_2_ after the modification and facilitates the subsequent binding with metal ions. CuS nanoparticles (NPs) were selected to improve the catalytic performance of PNSs/Ti_3_C_2_ hybrid materials. As a common metal, copper ion has good electrical conductivity and catalytic performance. It has been widely used in electrochemical sensors, carbon enrichment products, and hydrogen evolution reactions [29,30]. Additionally, sulfides are important semiconductor materials due to their metal-like properties for chemical sensing, and it should be noted that there is an additional advantage of cost-effectiveness of copper sulfide due to its Earth abundant property [31,32].

**Scheme 1:**
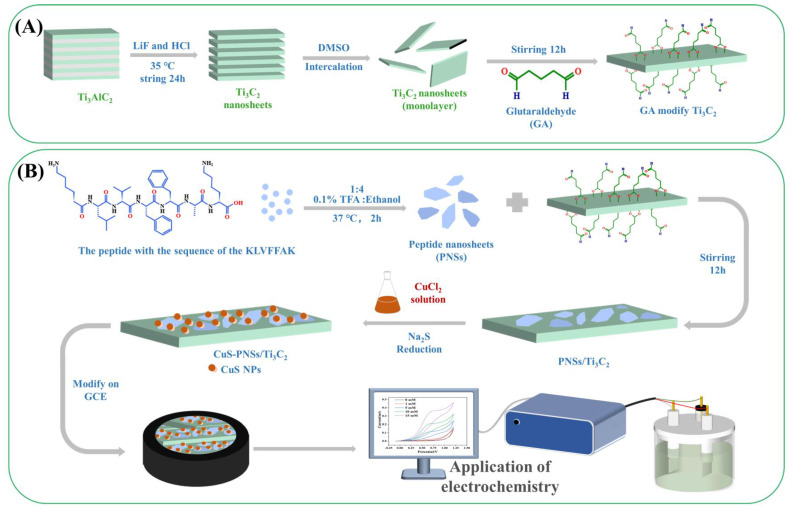
Schematic presentation of the biomimetic synthesis of CuS-PNSs/Ti_3_C_2_ and the fabrication of electrochemical H_2_O_2_ biosensors: (**A**) preparation of Ti_3_C_2_ nanosheets; (**B**) preparation of CuS-PNSs/Ti_3_C_2_ and fabrication of electrochemical H_2_O_2_ biosensors.

As shown in Scheme 1, the synthesized Ti_3_C_2_ nanosheets are cross-linked with self-assembled PNSs by GA linking to form the PNSs/Ti_3_C_2_ nanohybrids with abundant negative charges on the surface. Then, the adsorption of Cu^2+^ is achieved by electrostatic interaction between PNSs and Cu^2+^, and the in situ biomimetic synthesis of CuS NPs on the prepared PNSs/Ti_3_C_2_ nanohybrids is carried out by adding Na_2_S as the reaction agent, which is a green and friendly biomimetic synthesis method for the CuS-PNSs/Ti_3_C_2_ nanohybrids. Further, the created CuS-PNSs/Ti_3_C_2_ nanohybrids are modified onto the surface of GCE to construct the electrochemical H_2_O_2_ sensor platform. The fabricated biosensor platform exhibits good stability and high sensitivity, with a detection range of 5 μM–15 mM and a minimum detection limit of 0.226 μM. As an economic, highly catalytic, and electroactive material for detecting H_2_O_2_, the CuS-PNSs/Ti_3_C_2_-based electrochemical sensor platform has the advantages of excellent sensitivity, extremely high interference immunity, and long-term stability, showing potential applications for high-performance determination of H_2_O_2_ in biological and natural environments.

## 2. Materials and Methods

### 2.1. Materials and Reagents

A peptide with the sequence of KLVFFAK was bought from SynPeptide Biotechnology Co., Ltd. (Nanjing, China). Copper chloride, sodium sulfide, ethylene diamine tetracetic acid (EDTA), dimethyl sulfoxide (DMSO), Nafion, lysine (Lys), L-ascorbic acid (AA), and uric acid (UA) were obtained from Macklin Biochemical Co., Ltd. (Shanghai, PR China). Ethanol, hydrochloric acid, and sodium hydroxide (96%) were provided by Shanghai Test Laboratory Equipment Co., Ltd. (Shanghai, China). Lithium fluoride (LiF), titanium aluminum carbide (Ti_3_AlC_2_), sodium bicarbonate, and trifluoroacetic acid (TFA) were bought from Shanghai Yien Chemical Technology Co., Ltd. (Shanghai, China).

### 2.2. Synthesis of Ti_3_C_2_ Nanosheets

In order to obtain Ti_3_C_2_ monolayered nanosheets, the methods of HF etching of Ti_3_AlC_2_ and DMSO intercalation of multilayer Ti_3_C_2_ nanosheets were used to synthesize the Ti_3_C_2_ monolayer nanosheets on the basis of a previously reported method [33,34]. In brief, 1 mg LiF was dissolved in 20 mL HCl (12 M) for 10 min with magnetic stirring. Next, 1 mg Ti_3_AlC_2_ was added and magnetically stirred at 35 °C in a water bath for 24 h. Then, the black Ti_3_C_2_ solution was first washed with HCl (1 M) to remove the unreacted LiF and other impurities before being washed by deionized water at 8000 rpm rotating speed for 5 min until the final pH of the solution reached 6.0–7.0. Monolayer Ti_3_C_2_ nanosheets were obtained by sonicating the obtained solution for 30 min, and the middle part of the supernatant was collected for morphological characterizations. Clay-like multilayer Ti_3_C_2_ on the bottom of the centrifuge tubes was added into DMSO to intercalate under magnetic stirring overnight. To remove DMSO, a dialysis operation was applied. The sodium ascorbate solution was used as the exchange solvent to exchange DMSO for 24 h. Finally, freeze-drying was used to obtain the monolayer Ti_3_C_2_ pounds for further use.

### 2.3. Tailoring the Self-Assembly of Peptides into PNSs

PNSs are created by the self-assembly method by controlling the reaction condition, and the creation of PNSs in the whole study followed the following procedure: 20 mg of peptide (KLVFFAK) was dissolved in 10 mL 0.1% TFA and ethanol solution with a volume ratio of 1:9. The obtained peptide solution with a concentration of 2 mg mL^−1^ was then incubated at 47 °C in a water bath for 2 h to obtain PNSs.

### 2.4. Synthesis of CuS-PNSs/Ti_3_C_2_ Nanohybrids

The synthesis of the CuS-PNSs/Ti_3_C_2_ nanohybrids followed the following procedure: 10 mg Ti_3_C_2_ pounds was dissolved in 5 mL sodium ascorbate solution. To further modify the surface of the Ti_3_C_2_ nanosheets, 0.1% GA was added into the Ti_3_C_2_ solution with a volume ratio of 1:10 to obtain the GA-modified Ti_3_C_2_ under stirring for 12 h. After removing the uncombined GA through centrifuging, 2 mg mL^−1^ GA-modified Ti_3_C_2_ solution was mixed with prepared PNSs in the same volume under stirring for 12 h to obtain the PNSs/Ti_3_C_2_ nanohybrids. After 12 h reaction, a centrifuge was applied to remove the PNSs that did not combine with Ti_3_C_2_ nanosheets. After that, CuCl_2_ (10 mM) solution was added into PNSs/Ti_3_C_2_ solution with the volume ratio of 1:5 under stirring for 12 h, and Na_2_S was used to react with Cu^2+^ with the same volume to obtain the CuS-PNSs/Ti_3_C_2_ nanohybrids.

### 2.5. Electrochemical Detection of H_2_O_2_

GCEs (4.0 mm in diameter) were first polished with 0.3 mm and 0.05 mm alumina powder and then ultrasonically cleaned in ultrapure water and ethanol solution. The materials for the modification of GCEs were prepared by mixing 50 µL Nafion solution with 1 mL of PNSs, PNFs/Ti_3_C_2_, and CuS-PNSs/Ti_3_C_2_ solutions, respectively. After that, 10 mL of the modification solution was added dropwise onto the surface of GCE to obtain the Ti_3_C_2_/GCE, PNSs/Ti_3_C_2_/GCE, and CuS-PNSs/Ti_3_C_2_/GCE for subsequent electrochemical tests.

### 2.6. Characterization Techniques

All atomic force microscope (AFM) samples were prepared by dropping 10 mL of sample solution onto freshly cleaved mica substrates and air-dried for characterization. AFM measurements were performed in air using the FM-Nanoview 6800 AFM (FSM-Precision, Suzhou FSM Precision Instrument Co., Ltd., Jiangshu, China) using the tapping mode. Tap300Al-G (300 kHz, 40 N m^−1^) silicon probes were used for capturing AFM images. The tapping mode images were recorded and analyzed with Gwyddion software (Version 2.57). A transmission electron microscope (TEM, Tecnai G2 F20, FEI Co; Tokyo, Japan) was used to observe the structure and morphology of various nanocomposites. A scanning electron microscope (SEM, Regulus 8100, Hitachi, Japan) was used to observe the microstructure of the peptide-based nanocomposites. X-ray spectroscopy (XPS) characterization of the samples was performed on a PHI 5000 VersaProbe III spectrometer (UlVAC-PHI Company, Tokyo, Japan). All the electrochemical experiments were carried out at room temperature using an electrochemical workstation (CHI660E, Shanghai Chenhua, China) using a traditional three-electrode system, in which the working electrode was the modified GCE, the auxiliary electrode was platinum wire, and the reference electrode was a saturated calomel electrode.

## 3. Results

### 3.1. Characterizations of Ti_3_C_2_ and PNSs

**Figure 1:**
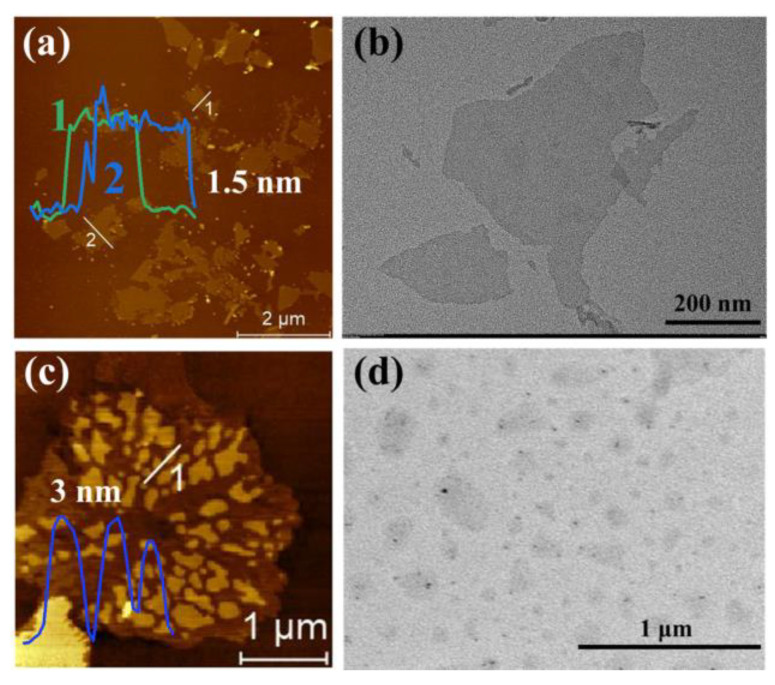
Morphological characterizations of Ti_3_C_2_ nanosheets and self-assembled PNSs: (**a**) AFM image of Ti_3_C_2_ nanosheets, the number and color line in green and bule corresponding to the thickness of 1 and 2 samples respectively; (**b**) TEM image of Ti_3_C_2_ nanosheets; (**c**) AFM height image of PNSs; (**d**) TEM image of PNSs.

The AFM technique was first used to characterize the prepared Ti_3_C_2_ monolayered nanosheets and self-assembled PNSs. It can be seen from Figure 1a that the Ti_3_C_2_ nanosheets were successfully prepared by HF etching for 24 h in a 37 °C water bath. In the obtained AFM image, the height of the Ti_3_C_2_ nanosheets is measured to be about 1.5 nm, which reveals good height distribution and agrees well with the height data previously reported for the Ti_3_C_2_ monolayer. In the corresponding TEM image (Figure 1b), the Ti_3_C_2_ nanosheets can also be seen clearly, and the size of the Ti_3_C_2_ nanosheets is between 200 nm and 1 µm, which is similar to the size that is shown in the presented AFM image. Therefore, we suggest that both AFM and TEM images prove that the Ti_3_C_2_ monolayered nanosheets with good distribution and uniform size were synthesized successfully.

In previous studies, it has been reported that the peptide with the sequence KLVFFAK could self-assemble into 2D peptide nanoribbons (PNRs) with a width of 200 nm and a length of more than 1 µm [35,36]. If the PNRs are used to modify Ti_3_C_2_ nanosheets, the size of the PNRs is bigger than that of the Ti_3_C_2_ nanosheets, which may cause the aggregation of Ti_3_C_2_ monolayers on the PNRs. Due to the aggregation of layers, the rate of electron movement of Ti_3_C_2_ multilayers is slower than that of Ti_3_C_2_ monolayers, which would significantly reduce the performance of electrochemical properties of peptide-based materials. Based on those studies, we tailored the self-assembly conditions of peptide molecules and synthesized PNSs with modernity size, which then can be modified onto the surface of Ti_3_C_2_ nanosheets for the formation of stable nanohybrids. As shown in Figure 1c, the formed self-assembled PNSs present a small size with a height of about 3 nm. The corresponding TEM image that is shown in Figure 1d indicates that the size of the formed PNSs is about 100–300 nm, which is much smaller than that of the Ti_3_C_2_ nanosheets, and could be suitable for the synthesis of Ti_3_C_2_ monolayer-based hybrid nanomaterials through GA linking.

In previous study, it has been found that the peptide with the KLVFFAK sequence can form nanostructures through the π–π interaction. Dai et al. found that the addition of inorganic salts would induce KLVFFAK to form large and thin nanobelts, and the size of nanobelts would increase with an increase in the salt concentration [37]. In the simulation study of Liang et al., the VFFA, KFFA, and FFFA sequences could self-assemble to form nanosheet structures, but VFFA, as the core of the KLVFFAK sequence, was less capable of forming large sheets than the other two sequences, and the VFFA sequence tended to form small nanosheet structures [38]. In the self-assembly system of this study, TFA can better dissolve the peptide powder, improve the dispersion of peptides in solution, and reduce the aggregation of polypeptide monomers, which makes it possible to form small-size nanostructures. At the same time, ethanol provides a more polar environment for the self-assembly of peptides, which tends to break the ring to form large-size nanosheets, thus facilitating the formation of small-size nanosheets.

### 3.2. Characterizations of CuS-PNSs/Ti_3_C_2_ Nanohybrids

The prepared PNSs and GA-modified Ti_3_C_2_ monolayered nanosheets were mixed together with the same volume at room temperature for 12 h under stirring to obtain the PNSs/Ti_3_C_2_ nanohybrids. Next, CuCl_2_ (10 mM) was added into the PNSs/Ti_3_C_2_ solution, and Cu^2+^ would be bound onto PNSs under the action of electrostatic adsorption between them. After 12 h stirring at room temperature, Na_2_S was added to react with Cu^2+^ for in-situ biomimetic synthesis of the CuS-PNSs/Ti_3_C_2_ nanohybrids.

**Figure 2:**
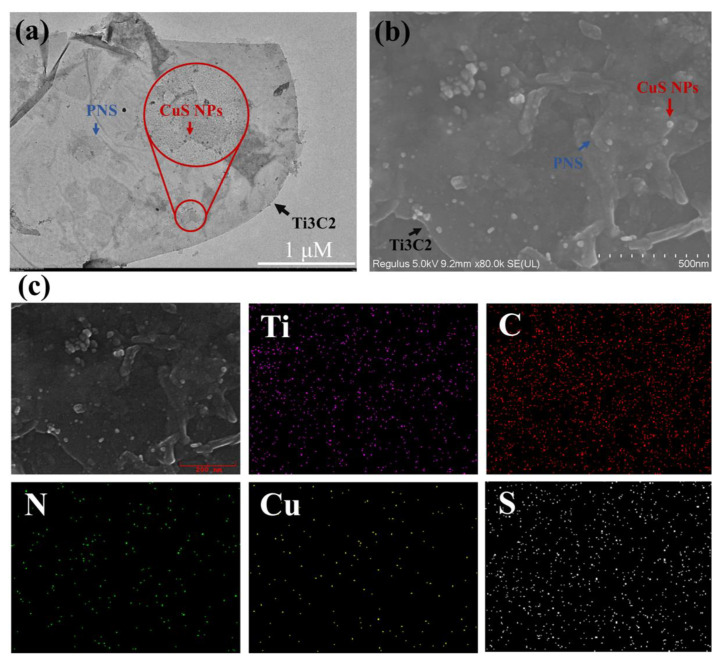
Characterizations of CuS-PNSs/Ti_3_C_2_ nanohybrids: (**a**) TEM image; (**b**) SEM image; (**c**) SEM-based elemental mapping.

Figure 2a provides the TEM image of the created CuS-PNSs/Ti_3_C_2_ nanohybrids, from which it can be seen that PNSs are conjugated onto the Ti_3_C_2_ monolayers, and a lot of CuS NPs are loaded onto the surface of the PNSs/Ti_3_C_2_ nanohybrids. Both SEM and elemental mapping were utilized to analyze the prepared CuS-PNSs/Ti_3_C_2_ nanohybrids. The corresponding SEM image in Figure 2b proves successful synthesis of CuS-PNSs/Ti_3_C_2_ nanohybrids, in which well-distributed CuS NPs are loaded on the surface of the PNSs/Ti_3_C_2_ nanohybrids. The formed PNSs and Ti_3_C_2_ nanosheets have the same appearance and there are fewer features to distinguish them; as a result of this, elemental mapping was applied to characterize the elemental distribution of the CuS-PNS/Ti_3_C_2_ nanohybrids. It can be observed that, besides Ti and C, the existence of N, Cu, and S provide strong evidence of the successful synthesis of the CuS-PNSs/Ti_3_C_2_ nanohybrids (Figure 2c).

**Figure 3:**
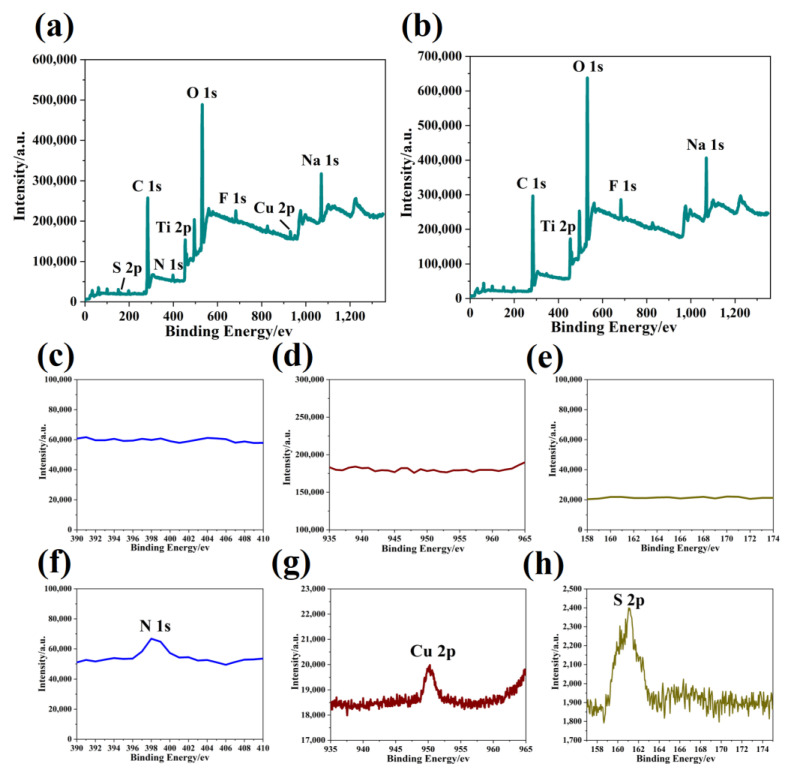
XPS characterization of CuS-PNS/Ti_3_C_2_ nanohybrids: (**a**) XPS survey spectrum of CuS-PNSs/Ti_3_C_2_; (**b**) XPS spectrum of Ti_3_C_2_; (**c**–**e**) XPS spectra of N, Cu, and S in Ti_3_C_2_; (**f**–**h**) XPS spectra of N, Cu, and S in the CuS-PNSs/Ti_3_C_2_ nanohybrids.

In order to identify the formation of CuS NPs and the combination of CuS NPs with PNSs and Ti_3_C_2_ into CuS-PNSs/Ti_3_C_2_, XPS characterization was used to measure the formed nanohybrids. The XPS spectrum of CuS-PNS/Ti_3_C_2_ clearly shows the existence of the peak of Ti2p, C1s, N1s, Cu2p, and S2p, as indicated in Figure 3a. Compared with this spectrum, the XPS survey spectrum of the prepared Ti_3_C_2_ nanosheets (Figure 3b) does not show the peak of N, Cu, and S, which further proves the synthesis of CuS-PNSs/Ti_3_C_2_. The fine XPS spectra shown in Figure 3c–e present the characteristic peaks of Ti_3_C_2_, in which the spectrum curves are flat without any clear characteristic peaks. However, after the formation of CuS-PNSs/Ti_3_C_2_, characteristic peaks are revealed at 398.3, 930.36, and 161.18 eV, which correspond to the typical peaks of N1s, Cu2p, and S2p, respectively (Figure 3f–h). In the process of Ti_3_C_2_ synthesis, HF etching and sodium ascorbate protection result in the binding of F^−^ and Na^+^ onto the surface of the Ti_3_C_2_ monolayer, which results in the appearance of the peaks of F1s and Na1s in the XPS spectra (Figure 3a,b).

Based on the above AFM, TEM, SEM, and XPS analysis, it can be concluded that the CuS-PNSs/Ti_3_C_2_ nanohybrids have been synthesized successfully via the biomimetic synthesis method.

### 3.3. CuS-PNSs/Ti_3_C_2_ Nanohybrid-Based Electrochemical Detection of H_2_O_2_

**Figure 4:**
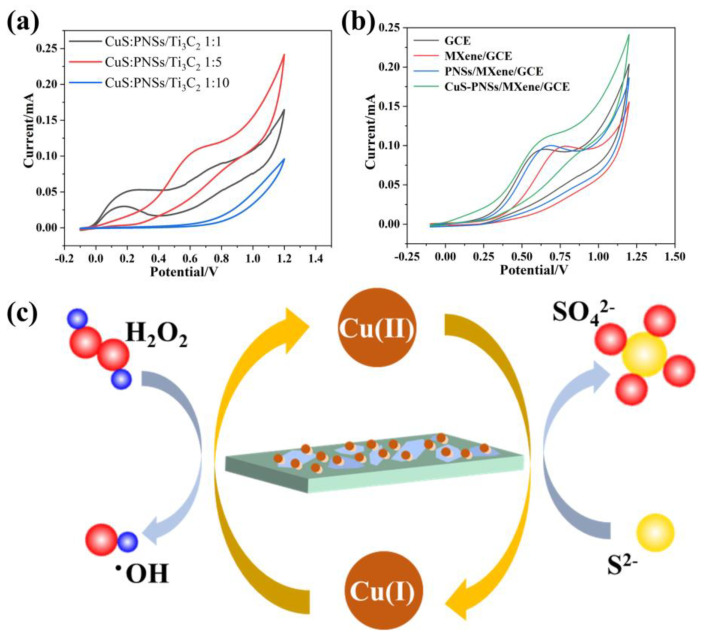
Electrochemical tests of different GCEs. (**a**) CV curves of different ratios of CuS and PNSs/Ti_3_C_2_ in the H_2_O_2_ concentration of 5mM; (**b**) CV curves of GCE, Ti_3_C_2_/GCE, PNSs/Ti_3_C_2_/GCE, and CuS-PNSs/Ti_3_C_2_/GCE; (**c**) electrochemical oxidization–reduction reaction mechanism by CuS NPs.

Electrochemical tests of the synthesized CuS-PNS/Ti_3_C_2_ nanohybrids were carried out in 0.1 M NaOH electrolyte for H_2_O_2_ sensing. First, the electrochemical tests of CuS-PNSs/Ti_3_C_2_ hybrids containing different ratios of CuS NPs were carried out. When the concentration of H_2_O_2_ was 5 mM, the ratio of CuS to PNSs/Ti_3_C_2_ was 1:1, 1:5, and 1:10, and they were conducted with cyclic voltammograms (CV) tests. As can be seen from the CV curves, the hybrid material with a ratio of 1:5 has a good and stable current response, as shown in Figure 4a. Next, different materials were modified onto the surface of GCEs to prepare functional electrodes for subsequent CV tests.

Figure 4b shows the CV curves of the prepared GCE, Ti_3_C_2_/GCE, PNSs/Ti_3_C_2_/GCE, and CuS-PNSs/Ti_3_C_2_/GCE, respectively. It was found that the fabricated CuS-PNSs/Ti_3_C_2_/GCE revealed the strongest current signal in the four GCEs with the potential of 0.4–0.8 V. Due to the contribution of the addition of CuS NPs, the electrochemical capabilities of the CuS-PNSs/Ti_3_C_2_ nanohybrid were improved significantly. Meanwhile, the addition of PNSs enriched the functional groups on the Ti_3_C_2_ monolayer surface and provided more combined points for the formation of CuS NPs, which is more beneficial to catalyze the decomposition of H_2_O_2_ to generate corresponding current responses.

As a 2D material with fast electron transfer rate, MXene has good electrical conductivity, which lays a foundation for the electrochemical performance of the CuS-PNSs/Ti_3_C_2_ nanohybrid. The addition of PNSs improves the richness of the surface groups of Ti_3_C_2_, modifies the surface with only hydroxyl and oxygen, further improves the binding force of hybrid materials to metals, and provides abundant sites for the nucleation and growth of CuS NPs. After the addition of CuS NPs, the catalytic performance of the CuS-PNSs/Ti_3_C_2_ nanohybrid was greatly improved, which promoted the detection of H_2_O_2_. When it comes to the electrochemical capabilities of CuS-PNSs/Ti_3_C_2_, CuS NPs play an important role in electrochemical reactions. As indicated in Figure 4c, Cu(II) is first reduced by S^2−^ into Cu(I), which meanwhile can reduce H_2_O_2_ to generate electrical signals. Combined with Figure 4a, it can be found that there is a weak current response between 0–0.25 V in the CV curve of the CuS-PNSs/MXene/GCE. We suggest that the weak current response here is attributed to the fact that, in the process of H_2_O_2_ dripping, S^2−^ in CuS NPs is first oxidized to SO_4_^2−^, and then the whole reduction process of H_2_O_2_ takes place.

**Figure 5:**
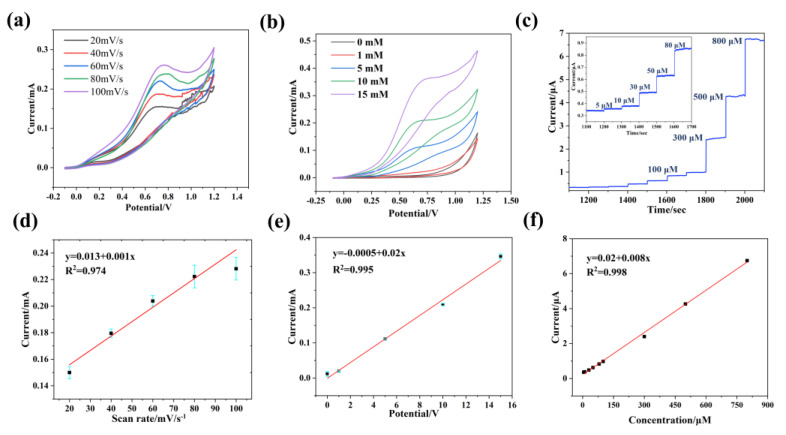
Electrochemical tests of CuS-PNSs/Ti_3_C_2_/GCE in H_2_O_2_ detection: (**a**) current response of CuS-PNSs/Ti_3_C_2_/GCE toward H_2_O_2_ with different scan rates of 20–100 mV s^−1^; (**b**) current response of CuS-PNSs/Ti_3_C_2_/GCE toward H_2_O_2_ with different concentrations of 0–15 mM; (**c**) I–T response of different concentrations of H_2_O_2_; (**d**) linear calibration of CV response with different scan rates of 20–100 mV s^−1^; (**e**) linear calibration of CV response with H_2_O_2_ concentration from 0 to 15 mM; (**f**) linear calibration of I–T response with H_2_O_2_ concentration from 5–800 µM.

To further test the electrochemical capability of the CuS-PNSs/Ti_3_C_2_ nanohybrids, the CV test of the different concentrations of H_2_O_2_ (0–15 mM) was carried out. First, different scan rates were applied to the CuS-PNSs/Ti_3_C_2_/GCE to determine the best scan rate. As it can be seen in Figure 5a, with the increase in scan rate, the current response increases significantly. Figure 5d shows the linear fitting of 20–100 mV s^−1^, which presents the great relationship of 20–80 mV s^−1^. Due to the obtained result, the scan rate of 50 mV s^−1^ was chosen for a further CV test. From Figure 5b, it can be found that with the increase in the concentration of H_2_O_2_, the current response increases gradually. Figure 5e shows the linear fitting of 0–15 mM concentration H_2_O_2_, which presents a great relationship. In order to explore the detection limit of the fabricated H_2_O_2_ electrochemical biosensor, an I–T test was applied for the CuS-PNSs/Ti_3_C_2_/GCE. The current response peak, found from the CV curve in Figure 5a, is 0.67 V; therefore, 0.67 V was chosen as the potential for the I–T test. It can be seen from the current response for the continuous addition of H_2_O_2_ in Figure 5c, when the H_2_O_2_ concentration reaches 5 µM in the solution, that an obvious current signal is generated. More importantly, with the increase in the H_2_O_2_ concentration, the I–T curve generates current responses of different intensities. The linear relationship between the current and H_2_O_2_ concentration has a good fit, and the linear correlation coefficient R^2^ is 0.997, as shown in Figure 5f. After the calculation, the lowest detection limit of the CuS-PNSs/Ti_3_C_2_/GCE-based electrochemical biosensor exhibits a detection limit of 0.226 µM towards H_2_O_2_, and the linear detection range is in the concentration range of 5–800 µM.

### 3.4. Selectivity and Stability of CuS-PNS/Ti_3_C_2_ Electrochemical Platform

In actual samples, there are some other substances in the solution besides H_2_O_2_. The nanohybrid-based electrochemical biosensors need to have good selectivity and be able to detect H_2_O_2_ sensitively among many interfering substances. Therefore, the selectivity of the CuS-PNSs/Ti_3_C_2_-based electrochemical biosensor was tested.

**Figure 6:**
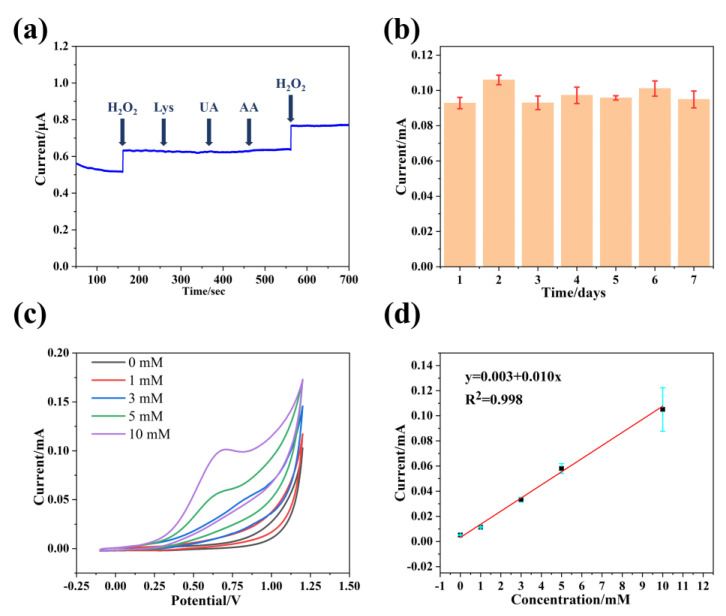
Anti-interference ability and stability of CuS-PNSs/Ti_3_C_2_-based electrochemical platform. (**a**) Amperometric responses upon successive additions of H_2_O_2_, Lys, UA, AA, and H_2_O_2_; (**b**) stability of CuS-PNSs/Ti_3_C_2_ electrochemical platform for 7 days; (**c**) current response of CuS-PNSs/Ti3C2/GCE toward H_2_O_2_ with different concentrations of 0–15 mM in milk; (**d**) linear calibration of CV response with H_2_O_2_ concentration from 0 to 15 mM in milk.

As shown in Figure 6a, when lysine, UA, and AA were added successively during the test, the I–T curve does not reveal an obvious current response; but when H_2_O_2_ is added again, the current intensity shows a very obvious current response. Therefore, it can be concluded that the CuS-PNSs/Ti_3_C_2_ hybrid material-based electrochemical biosensor has good anti-interference ability. Furthermore, to test the stability of the material, CuS-PNSs/Ti_3_C_2_/GCE was stored at −4 °C for 7 days, and the electrochemical detection ability of CuS-PNSs/Ti_3_C_2_/GCE for H_2_O_2_ was tested once every day. After seven days of testing, it was found that the electrochemical performance of the material was basically the same (Figure 6b), which indicates that the CuS-PNSs/Ti_3_C_2_/GCE has good stability and can still maintain an extremely high detection performance after long-term storage. At the same time, the actual sample was tested. H_2_O_2_ was added to the milk to prepare different concentrations of H_2_O_2_ milk solution to simulate the actual sample. In Figure 6c, it can be seen that the current response at 0.6–0.8 V increases with the increase in H_2_O_2_ concentration in milk, indicating that the CuS-PNSs/Ti_3_C_2_-based electrochemical biosensor also has good detection performance in reagent samples. In Figure 6d, it can be seen that the CuS-PNSs/Ti_3_C_2_-based electrochemical biosensor has a good linear relationship with the detection of real samples, and it can be utilized as a reliable electrochemical detection platform for determining H_2_O_2_.

## 4. Discussion

In this study, monolayered Ti_3_C_2_ MXene nanosheets with good morphology and good structure were synthesized by HF etching and DMSO intercalation. At the same time, in view of the fact that the edge of Ti_3_C_2_ is easy to oxidize, DMSO is replaced by sodium ascorbate by dialysis to protect Ti_3_C_2_, which slows down the rate of oxidation and prolongs the shelf life, addressing the problem that Ti_3_C_2_ is easily oxidized and decomposed into titanium dioxide in air. At the same time, in order to increase the richness and biocompatibility of Ti_3_C_2_ surface functional groups, PNSs were used to modify and functionalize the Ti_3_C_2_ surface. Through the design of the peptide sequence and the regulation of its self-assembly conditions, the PNSs with good morphology, moderate size, and negative charge in neutral condition were obtained. In order to realize the strong combination between Ti_3_C_2_ and PNS, GA was used as the bridge between them. The aldehyde group at one end of GA binds with the -OH group on the surface of Ti_3_C_2_, and the other end binds with the -NH_4_ group of PNSs to realize the recombination between them. Finally, to further improve the electrochemical performance of the materials, Na_2_S was used for in situ chemical reaction with Cu^2+^ to one-step synthesize CuS NPs without using additional reagent and waste production, meanwhile greatly improving the chemical properties of the CuS-PNSs/Ti_3_C_2_ nanohybrid materials.

Ti_3_C_2_ with large specific surface area can be loaded with PNSs on a large scale, which provides abundant binding sites for the nucleation and growth of CuS NPs. The one-step synthesis of CuS NPs on the surface of PNSs/Ti_3_C_2_ by direct reaction of sodium sulfide to prepare CuS-PNSs/Ti_3_C_2_ hybrid materials is a green, convenient, and environment-friendly biomimetic synthesis method. By using biomolecules as bridges and in-situ chemical reaction to synthesize sulphide NPs, the combination of metal sulfides and 2D materials is realized, which provides a new strategy and idea for the development of functional biosensors with good stability and selectivity. It has a broad prospect in biological detection, clinical application and environmental monitoring.

Based on the above AFM, TEM, SEM, and XPS analysis, it is confirmed that the CuS−PNSs/Ti_3_C_2_ nanohybrids have been synthesized successfully. At the same time, the biosensor platform has good electrochemical performance, anti−interference, and stability. Compared with another H_2_O_2_ detection platforms in Table 1, the CuS−PNSs/Ti_3_C_2_−based electrochemical biosensor platform in this study has a lower detection limit and a wider detection range of H_2_O_2_ concentration [39,40,41]. Therefore, in view of the wide detection range, low detection limit, and high sensitivity of the CuS−PNS/Ti_3_C_2_−based electrochemical sensors, it is suggested that it can be applied in the detection of H_2_O_2_ in a real environment, clinical detection, environmental protection, and biomedicine.

In the CuS−PNSs/Ti_3_C_2_ nanohybrids, the Ti_3_C_2_ nanosheets loaded with peptides provide binding sites for the formation of CuS NPs. At the same time, the existence of peptides promotes the dispersion of CuS NPs and prevents the performance degradation caused by the aggregation of NPs. In addition, for the synthesis of CuS NPs, a green and environment−friendly biomimetic synthesis method was adopted, and CuS NPs were formed in situ at the Cu^2+^ binding sites directly by using the self−assembled PNSs as templates. Compared with the traditional methods for CuS synthesis, such as the microwave−assisted solvothermal, chemical coprecipitation, and sol−gel synthesis, biomimetic synthesis in this current study is greener and more environmentally friendly, and there is no extra solvent and no additional waste liquid [42,43,44,45,46]. Furthermore, biomimetic CuS NPs can increase their biocompatibility to a certain extent and have more advantages for biological applications.

## 5. Conclusions

In summary, we report the peptide-medicated biomimetic synthesis of CuS NPs in hybrid 2D materials for the preparation of biosensors for the electrochemical detection of H_2_O_2_. The combination of Ti_3_C_2_ and PNSs and the modification of CuS NPs were realized by biological cross-linking and biomimetic synthesis, and CuS-PNSs/Ti_3_C_2_ nanohybrids were successfully prepared. The obtained results shows that CuS-PNSs/Ti_3_C_2_ nanohybrids have good sensitivity, high selectivity, and stability for the detection of H_2_O_2_. It is expected the design and synthesis of peptide nanomaterials for the binding with 2D materials and subsequent biomimetic synthesis functional nanomaterials could greatly enhance the properties and functions of hybrid nanomaterials and promote their application in various fields.

## Figures and Tables

**Table 1:** Comparison of the sensing performances of various electrochemical H_2_O_2_ sensors.

Materials	Linear Range [mM]	Limit of Detection [µM]	Ref.
CMF/Gox/HRP@MS	0.0001–0.235	10	[42]
rGO/AgNPs	0.002–20	0.73	[43]
HRP/Au/ERGO-CHIT/GCE	0.01–6.31	4	[44]
Cu-MOF/MXene	0.001–6.12	0.35	[45]
CuO-CeO2/MXene	0.005–0.1	1.67	[46]
CuS-PNS/Ti_3_C_2_	0.005–15	0.226	This work

## Data Availability

Not applicable.
